# Supplementary material for: Influence of Stress Severity on Contextual Fear Extinction and Avoidance in a Posttraumatic-like Mouse Model
Source: Brain Sci. 2024 Mar 26;14(4):311. doi: 10.3390/brainsci14040311 (PMC11048507; doi:10.3390/brainsci14040311)
Supplement: Supplementary file 1 [file brainsci-14-00311-s001.zip › brainsci-2918806-supplementary.pdf]

brain sciences

MDPI

*Supplementary Materials*

# Influence of Stress Severity on Contextual Fear Extinction and Avoidance in a Posttraumatic-Like Mouse Model

**Noémie Eyraud [1], Solal Bloch [1], Bruno Brizard [1], Laurane Pena [1], Antoine Tharsis [1], Alexandre Surget [1], Wissam El-Hage [1,2] and Catherine Belzung [1,*]**

[1] Institut National de la Santé et de la Recherche Médicale (INSERM), Imaging Brain & Neuropsychiatry iBraiN U1253, Université de Tours, 37032 Tours, France
[2] Pôle de Psychiatrie et d'Addictologie, Centre Hospitalier Régional Universitaire de Tours, 37000 Tours, France
[*] Correspondence: catherine.belzung@univ-tours.fr

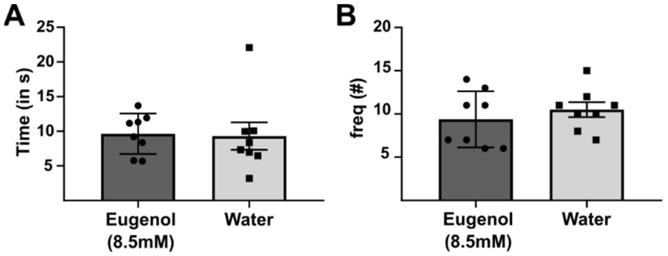


**Figure S1.** Eugenol did not induce preference behaviors in naïve C57BL6N mice. The odor preference test was performed in a three-chamber arena with on one side an object cleaned with an 8.5mM solution of eugenol and on the other side the identical object but cleaned with water. Mice were placed at the center of the arena and were allowed to explore for 10 minutes (A) the time spent sniffing the objects was manually measured. Mice spent the same amount of time sniffing both the eugenol-scented object and the neutral-scented object (paired t-test: t=0.1394, p=0.8930). (B) They did not exhibit higher frequency (freq) of smelling event on either object (paired t-test: t=0.6854, p= 0.5151).

**Table S1.** Detailed statistical analysis and results for each behavioral outcome presented (ext: Extinction).

| Behavioral Test | Statistical Test | p-Value | *post-hoc* |
|---|---|---|---|
| Distance moved during habituation to NOR arena | Shapiro-Wilk | p=0.1986 | |
| | Bartlett's | p=0.3070 | |
| | Brown-Forsythe | p=0.1238 | |
| | One-way ANOVA | F(2, 55)=10.58; p=0.0001 | Tukey<br>CTRL vs PTSD2: p=0.0004<br>CTRL vs PTSD4: p=0.0007<br>PTSD2 vs PTSD4: p=0.9786 |
| Correlation distance moved during habituation and freezing at the 1st re-exposure | Shapiro-Wilk | PTSD2:<br>Distance: p=0.5666; freezing: p=0.7867<br>PTSD4:<br>Distance: p=0.1865; freezing: p=0.6214 | |
| | Pearson | PTSD2: r=-0.3551; p=0.1261<br>PTSD4: r=-0.5422; p=0.0135 | |
| Familiarization during NOR learning | Shapiro-Wilk | p=0.0159 | |
| | Bartlett's | p=0.6741 | |

| | | | |
|---|---|---|---|
| | Kruskal-Wallis | H=11.39, p=0.0034 | Dunn<br>CTRL vs PTSD2: p=0.0042<br>CTRL vs PTSD4: p=0.0277<br>PTSD2 vs PTSD4: p>0.9999 |
| Distance moved during NOR test | Shapiro-Wilk | p=0.8745 | |
| | Bartlett's | p=0.1472 | |
| | Brown-Forsythe | p=0.1221 | |
| | One-way ANOVA | F(2, 55)=2.221; p=0.1182 | Tukey<br>CTRL vs PTSD2: p=0.9916<br>CTRL vs PTSD4: p=0.0545<br>PTSD2 vs PTSD4: p=0.0345 |
| NOR test (gross) | 2w RM ANOVA | Interaction: F(2, 55)=1.231; p=0.2999<br>grp: F(1, 55)=2.706; p=0.1057<br>obj: F(2, 55)=4.819; p=0.0118 | Sidak on obj factor new vs known<br>CTRL: p>0.999<br>PTSD2: p=0.9041<br>PTSD4: p=0.0868 |
| NOR test (normalized) | Shapiro-Wilk | p=0.9820 | |
| | Bartlett's | p=0.3019 | |
| | Brown-Forsythe | p=0.5024 | |
| | 1w ANOVA | F(2, 55)=4.113; p=0.0216 | |
| | One sample t test vs 50% | CTRL: t=0.05118; df=17; p=0.9598<br>PTSD2: t=0.1173; df=19; p=0.9078<br>PTSD4: t=3.539; df=19; p=0.0022 | |
| Distance moved during the avoidance test | Shapiro-Wilk | p=0.0004 | |
| | Bartlett's | p=0.0149 | |
| | Kruskal-Wallis | H=10.21; p=0.0061 | Dunn<br>CTRL vs PTSD2: p=0.0133<br>CTRL vs PTSD4: p=0.0187<br>PTSD2 vs PTSD4: p>0.9999 |
| Time spent on each object during avoidance test pre-ext | 2w RM ANOVA | Interaction: F(2, 55)=2.352; p=0.1047<br>grp: F(2, 55)=26.25; p<0.0001<br>obj: F (1, 55) = 23.28; p<0.0001 | Tukey (group factor):<br>CTRL vs PTSD2: p<0.0001<br>CTRL vs PTSD4: p<0.0001<br>PTSD2 vs PTSD4: p=0.8335<br>Sidak (obj factor: neutral - reminder):<br>CTRL: p=0.6481<br>PTSD2: p=0.0003<br>PTSD4: p=0.0077 |
| Time spent in each room during avoidance test pre-ext | 2w RM ANOVA | Interaction: F(2, 55)=12.83; p<0.0001<br>grp: F(2, 55)=18.67; p<0.0001<br>room: F(1, 55)=55.29; p<0.0001 | Tukey (interaction):<br>neutral: CTRL vs PTSD2: p=0.9891<br>CTRL vs PTSD4: p=0.9942<br>PTSD2 vs PTSD4: p=0.9660<br>reminder:CTRL vs PTSD2: p<0.0001<br>CTRL vs PTSD4: p<0.0001 |

| | | | |
|---|---|---|---|
| | | | PTSD2 vs PTSD4: p=0.5417<br>Sidak (neutral vs reminder)<br>CTRL: p=0.9987<br>PTSD2: p<0.0001<br>PTSD4: p<0.0001 |
| Extinction acquisition | RM GLM | Day×Time×group: F(12, 330)=1.28; p=0.2261<br>Day×group: F(4, 110)=21.22; p<0.0001<br>Time×group: F(6, 165)=15.82; p<0.0001 | Tukey (Day×group):<br>CTRL: day 1 vs day 2 p=0.8741<br>day 2 vs day 3 p>0.9999<br>PTSD2: day 1 vs day 2 p<0.0001<br>day 2 vs day 3 p=0.0002<br>PTSD4: day 1 vs day 2 p<0.0001<br>day 2 vs day 3 p=0.0006<br>Day 1: CTRL vs PTSD2 p<0.0001<br>CTRL vs PTSD4 p<0.0001<br>PTSD2 vs PTSD4 p=0.9320<br>Day 2: CTRL vs PTSD2 p<0.0001<br>CTRL vs PTSD4 p<0.0001<br>PTSD2 vs PTSD4 p=0.9925<br>Day 3: CTRL vs PTSD2 p<0.0001<br>CTRL vs PTSD4 p<0.0001<br>PTSD2 vs PTSD4 p=0.8312<br>Tukey (time×group):<br>CTRL: 5min vs 10min p>0.9999<br>5min vs 15min p>0.9999<br>5min vs 20min p>0.9999<br>PTSD2 : 5min vs 10min p=0.8679<br>5min vs 15min p<0.0001<br>10min vs 20min p<0.0001<br>PTSD4 : 5min vs 10min p>0.9999<br>5min vs 15min p<0.0001<br>10min vs 20min p<0.0001 |
| Retrieval vs 1st re-exposure | 2-way ANOVA | Interaction: F(2, 55)=13.24; p<0.0001<br>grp: F(2, 55)=115.3; p<0.0001<br>day: F(1, 55)=137.3; p<0.0001 | Tukey (retention):<br>CTRL vs PTSD2: p<0.0001<br>CTRL vs PTSD4: p<0.0001<br>PTSD2 vs PTSD4: p=0.6361<br>Sidak (day factor: re-expo 1 - retention):<br>CTRL: p=0.0435<br>PTSD2: p<0.0001<br>PTSD4: p<0.0001 |
| % of extinction: PTSD2 vs PTSD4 | Shapiro-Wilk | p=0.2315 | |
| | F test | p=0.1328 | |
| | Unpaired t-test | p=0.3613 | |
| Proportion of low extinction | Khi2 | P=0.52 | |
| Distance moved during the avoidance test post-extinction | Shapiro-Wilk | p=0.2863 | |
| | Bartlett's | p=0.8603 | |
| | Brown-Forsythe | p=0.7180 | |
| | 1w ANOVA | F(2, 27)=7.715; p=0.0022 | Tukey<br>CTRL vs PTSD2: p=0.0016<br>CTRL vs PTSD4: p=0.0652<br>PTSD2 vs PTSD4: p=0.2861 |

| | | | |
|---|---|---|---|
| Time spent on each object during avoidance test post-ext | 2w RM ANOVA | Interaction: F(2, 27)=6.899; p=0.0038<br>grp: F(2, 27)=2.995; p=0.0669<br>obj: F(1,27)=4.599; p=0.0411 | Tukey (interaction):<br>neutral:<br>CTRL vs PTSD2: p=0.8097<br>CTRL vs PTSD4: p=0.0337<br>PTSD2 vs PTSD4: p=0.1334<br>reminder:<br>CTRL vs PTSD2: p=0.0033<br>CTRL vs PTSD4: p=0.0870<br>PTSD2 vs PTSD4: p=0.4040<br>Sidak (neutral vs reminder)<br>CTRL: p=0.2417<br>PTSD2: p=0.0684<br>PTSD4: p=0.0141 |
| Time spent in each room during avoidance test post-ext | 2w RM ANOVA | Interaction: F(2, 27)=7.020; p=0.0035<br>grp: F(2, 27)=2.641; p=0.0896<br>obj: F(1,27)=27.36; p<0.0001 | Tukey (interaction):<br>neutral: CTRL vs PTSD2: p=0.0765<br>CTRL vs PTSD4: p=0.0689<br>PTSD2 vs PTSD4: p=0.9988<br>reminder: CTRL vs PTSD2: p=0.0005<br>CTRL vs PTSD4: p=0.0038<br>PTSD2 vs PTSD4: p=0.7997<br>Sidak (neutral vs reminder)<br>CTRL: p>0.9999<br>PTSD2: p=0.0002<br>PTSD4: p=0.0006 |
| Latency to go to the lighted room (BCO) | Shapiro-Wilk | p<0.0001 | |
| | Bartlett's | p<0.0001 | |
| | Kruskal-Wallis | H=1.456; p=0.4828 | |
| time spent in the lighted room (BCO) | Shapiro-Wilk | p<0.0001 | |
| | Bartlett's | p=0.0177 | |
| | Kruskal-Wallis | H=2.093; p=0.3512 | |
